# Improving STI Screening in Adolescent and Young Adult Men in a Primary Care Setting

**DOI:** 10.1097/pq9.0000000000000807

**Published:** 2025-05-14

**Authors:** Jessica Addison, Ramy Yim, Ben Ethier, Maria Alfieri, Lydia A. Shrier, Allison Pellitier, Susan Fitzgerald, Gabriela Vargas, Josh Borus

**Affiliations:** From the * Division of Adolescent/Young Adult Medicine, Boston Children’s Hospital, Boston, Mass.; † Harvard Medical School, Boston, Mass.; ‡ Department of Pediatrics Quality Program, Boston Children’s Hospital, Boston, Mass.

## Abstract

**Introduction:**

Adolescents and young adults (AYAs) account for approximately half of all new diagnoses of sexually transmitted infections (STIs) in the United States. Screening AYA men is imperative to stopping the spread of infection as well as preventing long-term sequelae. Although our AYA medical practice has consistently screened AYA women at rates more than 80% annually, the baseline screening rate for men was less than 70%.

**Methods:**

Between May 2021 and October 2023, we conducted a quality improvement initiative among male primary care patients older than 15 years who had an annual physical within the past 3 years. Interventions included adding a bathroom sign clearly stating urine would not be used for drug testing and creating and implementing a chlamydia and gonorrhea (GC/CT) testing alert in the electronic health record for all male medical visits. Our primary outcome was the percentage of patients who received GC/CT screening.

**Results:**

Statistical process control p-chart analysis showed special cause variation with improved GC/CT screening rates among AYA men in primary care, including a significant increase in the mean screening rate from 73.5% to 83.5% following our second intervention, demonstrating a mean shift from previous results.

**Conclusions:**

Clinic-level interventions—bathroom signage indicating urine would not be used for drug testing did not improve STI screening rates, whereas an electronic health record prompt for clinic staff regarding the need for STI testing—improved GC/CT screening rates among AYA men in primary care.

## INTRODUCTION

Adolescents and young adults (AYAs) account for approximately half of all new sexually transmitted infection (STI) diagnoses in the United States.^1^ In 2022–2023, chlamydia (CT) was the most frequently reported bacterial STI, followed by gonorrhea (GC). Youth 15–24 years of age accounted for 55.8% of reported CT infections and 48.4% of reported GC infections.^1–3^

Although there is insufficient evidence to recommend routine screening for GC/CT among sexually active AYA men because of concerns such as efficacy and cost-effectiveness, screening of sexually active AYA men should be considered in clinical settings with a high prevalence of STIs (eg, adolescent clinics, STI specialty clinics) or for populations with a high burden of infection (eg, men who have sex with men).^4,5^ Because the rate of positive chlamydia testing exceeds 5% (eg, 6.67% in 2022) among men in our population, our standard of care is to screen all sexually active AYA men annually.

Our AYA medical practice has consistently screened sexually active AYA men at annual rates more than 80%. Still, the baseline screening for males was approximately 70% at the start of the COVID-19 pandemic (March 2020) and as low as 58% at the height of the pandemic (November 2020). This project aimed to increase GC/CT screening in sexually active men older than 15 years who were AYA primary care patients, from the nadir level of 58% during the pandemic to at least 80% by July 2022 through trialing quality improvement interventions. Although full STI screening includes trichomonas, HIV, and syphilis, for this project, we focused on GC/CT screening, the most frequent infections.

## METHODS

**Fig. 1.**
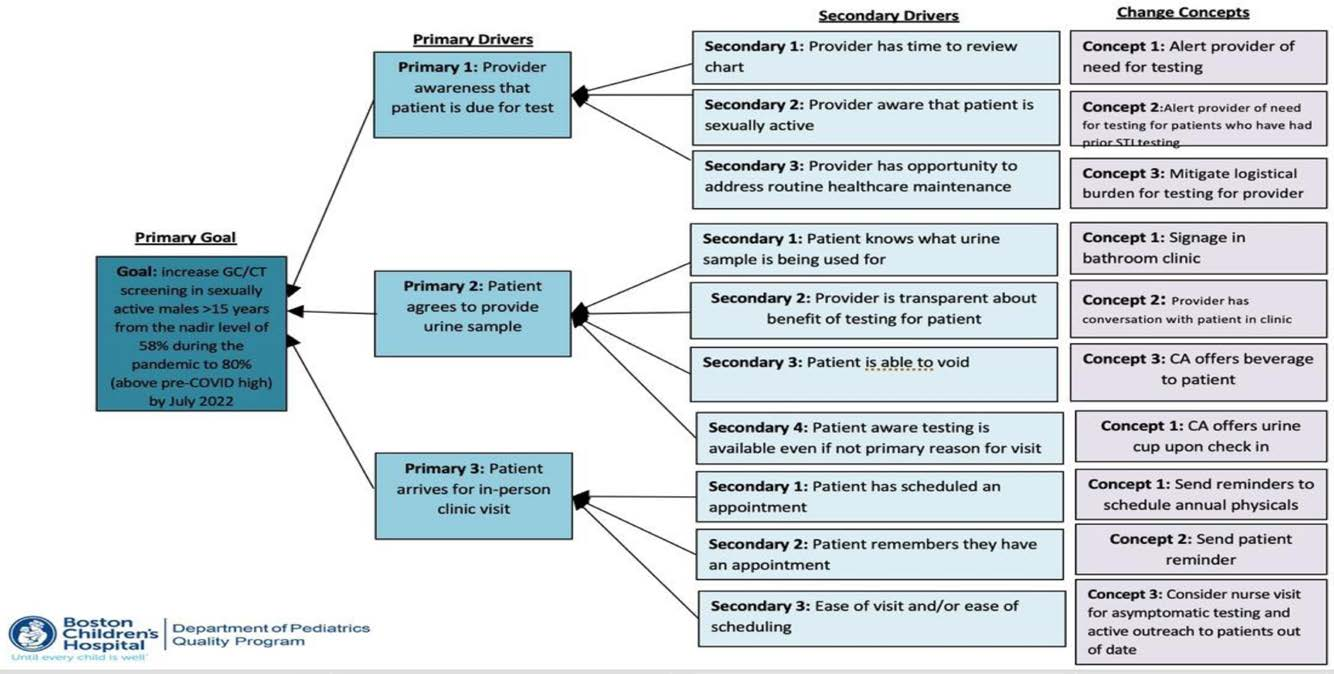
Cause of problem-driver diagram.

The project occurred in an AYA medicine clinical practice affiliated with an urban, tertiary care, academic, free-standing children’s hospital in the Northeast United States. This clinic is staffed by attending physicians, nurse practitioners, adolescent medicine fellows, and pediatric residents caring for patients 12–26 years of age. We created an STI Quality Improvement working group consisting of doctors, clinical assistants, nurses, administrators, and staff from our institution’s pediatric quality program. This group evaluated clinic workflow and used an Ishikawa diagram (**see Supplement 1, Supplemental Digital Content 1,** http://links.lww.com/PQ9/A652) to examine potential missed opportunities for GC/CT screening among male patients older than 15 years of age, using sex and age documented in the electronic health record (EHR). Subsequently, a key driver diagram was created to identify the primary and secondary drivers leading to change concepts that could address barriers to GC/CT testing (Fig. 1). The team used a division-wide meeting to discuss the interventions and guidelines for STI screening. This allowed for feedback from the entire community and facilitated the adoption of change. This project was reviewed by the institution’s Hospital Quality Improvement Committee, and considered it exempt from review by the institutional review board.

### Interventions

We focused on 2 interventions: (1) adding a bathroom sign clearly stating urine would not be tested without the patient’s consent to address concerns about drug testing and (2) creating and implementing a GC/CT testing alert in our EHR. This alert allowed us to identify male patients overdue for STI testing. A patient was considered overdue for testing if they had been tested previously but had not had repeat testing within the past 13 months. Interventions were tested using plan-do-study-act cycles and adopted after several tests of change. We implemented the initial intervention in May 2021 and the second in December 2021. “We reviewed patients” GC/CT results from October 2019 (pre-COVID-19) through October 2022 (including pre- and postintervention periods).

Despite our practice policy of not administering drug screens or any other testing without a patient’s explicit consent, we realized in discussion with patients that this remained a concern and resulted in patients declining to leave a urine sample. Clinical assistants asked patients to leave a urine sample upon check-in for all physical examination and certain urgent care visits (eg, dysuria). Still, patients were not always clear on the purpose of the sample. Therefore, the first intervention adopted was adding a prominently placed sign to all bathrooms clearly stating urine would not be tested without the patient’s consent. This was done to alleviate patients’ concerns regarding drug testing. Signage was in English and Spanish, the preferred language of more than 95% of our patient population.

Next, we developed the second intervention—creating and implementing a GC/CT testing alert for all male medical visits. Our EHR was mined via a programmable data pull on Monday mornings to generate a list of patients with visits that week who had had GC/CT testing in the past but not within the last 13 months, indicating they were overdue for annual STI screening. A note was manually added to “reason for visit” within a patient’s chart at the start of each week, indicating they were overdue for STI testing in addition to the primary reason for the visit. The reminder prompted clinical assistants to obtain a urine specimen and clinicians to offer GC/CT testing during the visit. When this was successful, we built a fully automated reminder into the EHR, prompting the provider to screen for GC/CT whenever the chart was opened.

### Study of the Intervention(s)

We reviewed medical records starting in October 2019 to identify eligible patients. Using an automated search of our EHR data, we identified all male patients older than 15 years of age with an annual physical in the past 3 years. We selected the 3-year timeframe because that is the practice standard for defining a current patient in our practice. Primary care patients are encouraged to have an annual physical examination, at which screening and testing for STIs is obtained if ever sexually active. We excluded AYA men receiving consultative care, as their STI screening is addressed by their primary care provider. Previous GC/CT testing was used as a marker for sexual activity, so patients with previous urine or urethral testing were identified as those requiring retesting. We conducted a thorough chart review using the outpatient electronic medical record, Cerner PowerChart (Kansas City, Mo.)^6^ to ensure patients were being correctly identified by the program. Thirty charts were randomly selected and reviewed by 2 coinvestigators, and differences were reconciled before completion of the review.

Preliminary data obtained between October 2019 and April 2021 served as our baseline data. The bathroom signage was implemented in May 2021, and the EHR alert was implemented in December 2021. We monitored data monthly to assess the impact of our interventions.

### Measures

We looked at 2 primary measures. First, the percentage of primary care male patients with GC/CT testing more than 13 months ago (sexually active male patients) who had a visit within the past year and had GC/CT testing was calculated to measure the impact of our intervention on our overall population’s testing status. We tracked monthly GC/CT testing rates at encounters where a male patient was identified as overdue for testing.

### Analysis

We used statistical process control charts to analyze changes in outcome measures. The percentage of eligible patients who had GC/CT screening obtained within 13 months of the previous visit were analyzed by using control charts. The control charts identified notable events that impacted care, such as supply chain issues, the COVID-19 pandemic, and the expansion of virtual visits that might have affected the normal distribution. Standard control chart rules were used to identify shifts in the mean. SQCPack version 7.0 (PQ Systems, Dayton, Ohio) was used to create the p-charts.

## RESULTS

**Fig. 2.**
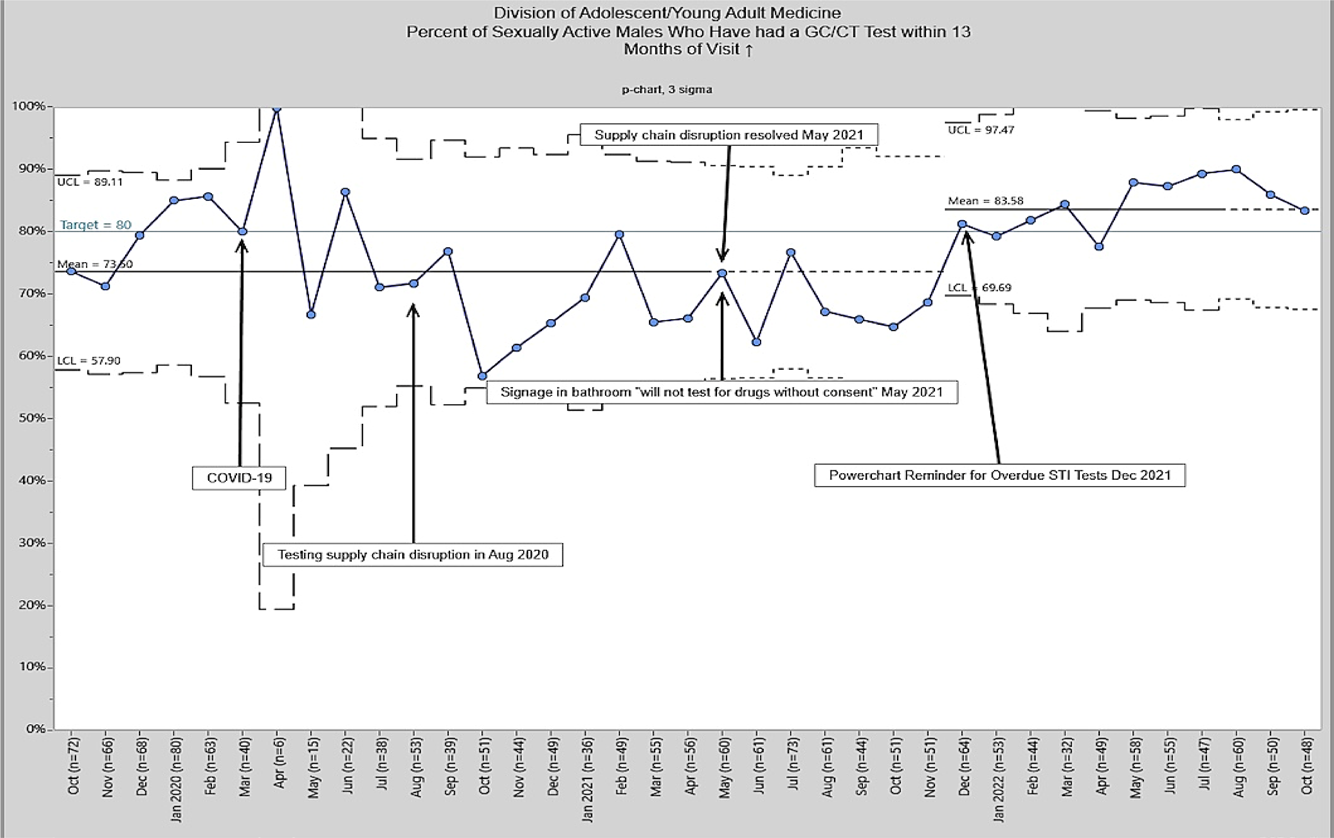
Percent of sexually active males due for GC/CT who were tested.

A total of 355 patients met the inclusion criteria. The majority of patients spoke English as their primary language (n = 306, 86.2%) and had public insurance (n = 198, 55.8%), whereas a plurality self-identified as African American (n = 172, 48.4%). Additional patient demographic data are listed in Table 1. Baseline data were from October 2020 to April 2021, when the mean screening rate was 73.6%. The bathroom signage intervention was implemented in May 2021. Our analysis showed no special cause variation, with no significant increase in the mean screening rate (Fig. 2). Following the EHR alert, GC/CT screening opportunities were evaluated at monthly intervals. We observed a significant shift in mean testing rates. There was an increase from 73.5% during the baseline period to 83.5% starting in December 2021 (Fig. 2).

**Table 1.** Demographic Characteristics of Primary Care Patients During the Intervention Period, Between May, 2021and October 2022

	Total (N)		Percentage
All subjects	355		100
Race and ethnicity			
White		45	12.7
Black or African American		172	48.4
Asian		6	1.7
Another race, non-Hispanic		32	9.0
Hispanic		79	22.3
Multiracial, non-Hispanic		4	1.1
Declined to answer/not specified		17	4.8
Primary language			
English	306		86.2
Non-English	49		13.8
Insurance type			
Private	138		38.9
Public	198		55.8
Not reported	19		5.3

## DISCUSSION

We implemented 2 quality improvement interventions to increase AYA men’ GC/CT screening rates. The first intervention was informed by the American Academy of Pediatrics recommendations against involuntary drug testing of teens, even with parental consent, unless there are substantial medical or legal reasons as well as patient input.^7^ When adolescents perceive that health care services are not confidential, reports indicate they are less likely to seek care, particularly for reproductive health matters or substance abuse.^8,9^ This intervention did not result in a mean shift in STI screening rates.

There was an improvement in screening rates with a shift in the mean after the initiation of our second intervention, the EHR reminder. Creating the alert visible to all relevant members of the care team (clinical assistants and medical providers) generated the opportunity for timely decision-making at all testing step processes. As it demonstrated impact, we moved from a reminder that required a low staff effort (approximately 20 min a week), which captured most opportunities for testing, to a completely automated reminder, which captured all opportunities for testing.

Despite recommendations from national medical organizations such as the American Academy of Pediatrics to screen adolescents for STIs, pediatricians do not routinely screen patients for a variety of reasons (eg, provider discomfort, patient confidentiality concerns, and time constraints).^10^ In a busy primary care setting where clinicians must quickly address many issues, it is easy to overlook STI screening in men, who are often asymptomatic and less likely to come for physical exams where screening is a priority. Our findings are consistent with a study showing that EHR reminders can improve STI screening rates among both male and female adolescents seen in a pediatric emergency department.^11^ In that study, patients who had a sexual health screener embedded in the EHR had 5 times greater odds of STI screening compared with patients who did not have the screener, which served as a prompt for providers.^11^

## LIMITATIONS

This was a single-center study at an urban teaching hospital, which impacts the findings’ generalizability. However, this study’s challenges might apply in other clinical settings. The COVID-19 pandemic introduced disruptions that impacted screening patterns and introduced confounding factors in this study. These external factors also contributed to variations in the baseline data before the start of the interventions. There was potential for missed opportunities for reminders that may have resulted in missed STI screening opportunities if administrative assistants made an appointment after the weekly screening. This was ultimately rectified by adopting a fully automated reminder. Our internal marker to determine if a patient has been sexually active (prior STI screening) has limitations. If a patient did not feel comfortable disclosing that they were sexually active, or if the provider did not ask about sexual activity, or if an STI order was not placed in a patient who reported being sexually active, they would not be included in our population of interest. Finally, our chart review was retrospective, so we could not always determine why GC/CT screening was not performed, which we counted as missed opportunities for testing.

## CONCLUSIONS

This quality improvement initiative was effective at improving STI screening rates in AYA men through the development and implementation of 2 interventions. Bathroom signage indicating patient’s consent would be obtained before any testing could be done; however, this did not improve screening rates. Importantly, EHR prompts to alert clinic staff to male patients overdue for STI screening did improve screening rates in a sustainable manner.

## Supplementary Material


