# Supplementary material for: Improving STI Screening in Adolescent and Young Adult Men in a Primary Care Setting
Source: Pediatr Qual Saf. 2025 May 14;10(3):e807. doi: 10.1097/pq9.0000000000000807 (PMC12077505; doi:10.1097/pq9.0000000000000807)
Supplement: Supplementary file 1 [file pqs-10-e807-s001.pdf]

Supplement 1: Cause and Effect Fishbone Diagram

Cause of the Problem-Cause & Effect (Fishbone Diagram)

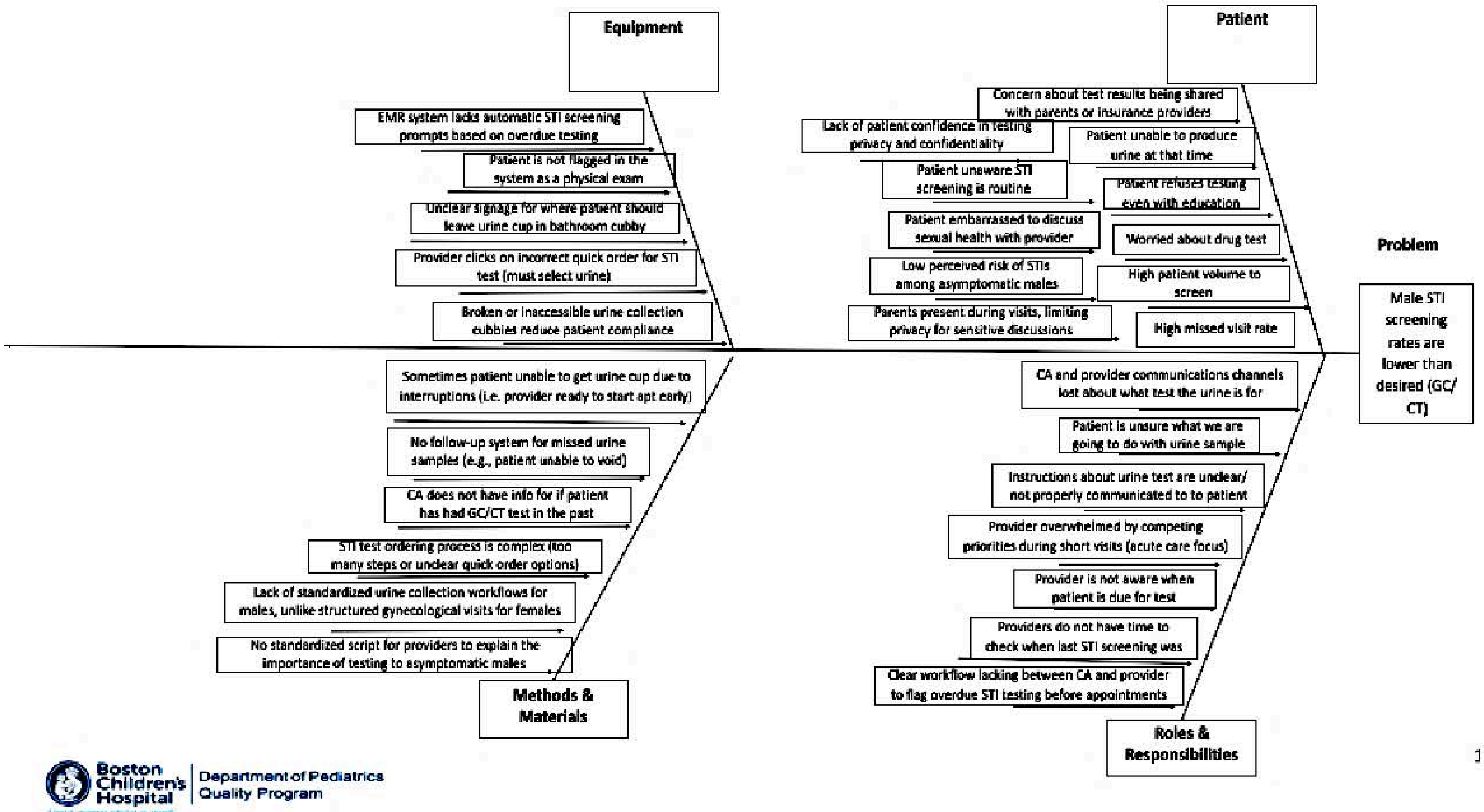

Boston Children's Hospital | Department of Pediatrics Quality Program
